# Enhanced Infant Movement Analysis Using Transformer-Based Fusion of Diverse Video Features for Neurodevelopmental Monitoring

**DOI:** 10.3390/s24206619

**Published:** 2024-10-14

**Authors:** Alexander Turner, Don Sharkey

**Affiliations:** 1 School of Computer Science, University of Nottingham, Nottingham NG8 1BB, UK; 2 Centre for Perinatal Research, School of Medicine, University of Nottingham, Nottingham NG7 2RD, UK; don.sharkey@nottingham.ac.uk

**Keywords:** neurological development, infant development, transformers, vision transformers, autonomous monitoring, movement assessment of infants, machine learning

## Abstract

Neurodevelopment is a highly intricate process, and early detection of abnormalities is critical for optimizing outcomes through timely intervention. Accurate and cost-effective diagnostic methods for neurological disorders, particularly in infants, remain a significant challenge due to the heterogeneity of data and the variability in neurodevelopmental conditions. This study recruited twelve parent–infant pairs, with infants aged 3 to 12 months. Approximately 25 min of 2D video footage was captured, documenting natural play interactions between the infants and toys. We developed a novel, open-source method to classify and analyse infant movement patterns using deep learning techniques, specifically employing a transformer-based fusion model that integrates multiple video features within a unified deep neural network. This approach significantly outperforms traditional methods reliant on individual video features, achieving an accuracy of over 90%. Furthermore, a sensitivity analysis revealed that the pose estimation contributed far less to the model’s output than the pre-trained transformer and convolutional neural network (CNN) components, providing key insights into the relative importance of different feature sets. By providing a more robust, accurate and low-cost analysis of movement patterns, our work aims to enhance the early detection and potential prediction of neurodevelopmental delays, whilst providing insight into the functioning of the transformer-based fusion models of diverse video features.

## 1. Introduction

The neurological development pathway, which encompasses the brain, nerves and spinal cord, is both complex and influenced by many factors. Neurological disorders in infants can present in diverse ways, but many which manifest are visible in early life [1,2,3]. Such disorders can have a significant impact on a person’s life and require long-term care and intervention. Movement disorders and delays in neurological development can lead to difficulties in performing daily activities, mobility issues, and diminished social interactions [4,5,6]. Early identification and intervention might harness the high neuroplasticity present in infants, optimising therapeutic outcomes [7,8].

There are many forms of neurological disorders which have physical manifestations, with one of the most prevalent being cerebral palsy (CP). CP primarily affects movement, coordination, balance, and walking [9] but can be associated with difficulties in speech, hearing, and vision. The condition arises due to injury or abnormal development to the infant’s neurological system either before, during, or shortly after birth, which can be caused by factors such as genetics, infections, stroke, head injuries, or oxygen deprivation. The prevalence of CP is approximately 2.1 per 1000 live births, with the risk notably higher in preterm infants [9,10]. Those born between at less than 23 weeks have a 4.6% chance of developing CP [11]. Early detection and intervention are critical in managing CP and improving the quality of life for affected individuals [12,13,14,15]. CP is typically diagnosed when developmental milestones, such as crawling and walking, are delayed. This makes early detection challenging, resulting in an average diagnosis age of between 12 and 24 months [13,14,15,16].

Infant motor development follows a predictable trajectory that offers important insights into neurodevelopmental progress. Between 4 and 8 months, infants typically master rolling from front to back and back to front, reflecting increased control over their movements. By 9 to 11 months, most infants can sit independently without support, showcasing enhanced postural control and coordination. Around 12 to 14 months, the ability to crawl usually develops, marking a major step forward in motor and cognitive coordination. These milestones, which emerge between 3 and 12 months, serve as critical indicators of healthy neurodevelopment and are vital for identifying early signs of delays or disorders, such as cerebral palsy. Monitoring these key stages can provide valuable opportunities for early intervention and better long-term outcomes [17,18].

To try and limit the impact of neurological disorders, there has been a push towards early diagnosis, with a particular focus on automation and the use of artificial intelligence to achieve this. There have been many different attempts, with many focusing on improving diagnostic accuracy and speed, providing healthcare professionals with better information [19,20,21]. Moreover, there have also been significant attempts to try and automate the Prechtl’s General Movements’ Assessment (GMA) [22,23,24]. The GMA has proven highly effective in classifying CP, with a sensitivity of 98% and a specificity of 91%. These works use pose estimation, the process of trying to capture and classify movement using a specific set of algorithm which track the positions and orientations of the human body from images, sensors and video. Moreover, the tools used to accomplish this have been evolving, and are commonly underpinned by deep learning models [25,26,27]. However, pose estimation algorithms can have performance reductions when limbs are obscured, especially when dealing with a single fixed-point camera and the variable orientations of infants.

Machine learning (ML) techniques have steadily improved, expanding possibilities for autonomous neurological disorder assessments. A key advancement is the use of transformer networks, initially developed for text processing but now widely applied in video classification, surpassing convolutional neural networks [28,29,30]. Another major development is the rise of multi-modal architectures, which integrate various data types (video, image, text, audio) into a single network, enhancing performance through complementary insights and greater robustness to noise and missing data. These innovations have led to significant improvements in fields like autonomous driving and healthcare [31,32].

By building upon previous work [33], this work aims to develop an accurate, robust, and low-cost open-source vision-based movement analysis pipeline that fuses video features from a pre-trained convolutional neural network, a pre-trained vision transformer, and a pose estimation algorithm within a transformer-based machine learning model to improve the detection and classification of infant movements. We hypothesize that this fusion of diverse video features will enhance classification accuracy and overcome challenges of pose estimation such as occluded limbs and unregistered poses, with sensitivity analysis revealing the most influential features in this process.

## 2. Materials and Methods

Twelve infants of mixed genders with no known neurological conditions, aged between 3 and 12 months, were placed on a play mat and recorded while playing organically with toys. This age range was chosen as it represents a critical period in infant development, during which key motor milestones typically emerge. Observing infants’ interactions with toys offers valuable insights into their motor and cognitive development, as play behaviour reflects their ability to explore, coordinate movements, and engage with their environment. Approval for this study was granted by the Department of Computer Science at Nottingham University (approval number: CS-2020-R-73). Each session was planned to last no longer than one hour per participant, though the actual recording times varied between 16 and 43 min depending on the infant’s mood and parental availability. Infants were placed on a play mat, allowing them to move freely while they interacted with various toys. To ensure accurate 2D pose estimation, recordings were made from a top-down perspective. A Canon EOS 70 digital video camera with a wide-angle lens (640 × 480 resolution) was used, capturing videos at 25 frames per second.

Three labels were derived to classify the dexterous movement of the infants when interacting with toys:

1. No control of any toy (NC).
2. Full control with a single hand (FC1H), defined as when an infant grasped the object and moved it of their own accord for a sustained period (approximately three seconds to differentiate from limited control).
3. Full control with two hands (FC2H), when the infant had grasped the object with both hands and manipulated it.

The data were also additionally labelled with limited control for both 1 and 2 hands; however, this frequency of movement was limited and therefore omitted from this work.

Data were extracted from raw videos according to the specific labels listed above. For each instance of each label, a video segment corresponding to the exact time point of that movement was extracted. Specifically, for each time point, a two-second video segment was extracted at 24 fps, spanning from one second before and after the labelled movement. Each video contains 49 frames.

To augment the dataset and maximize the data available for training the classifiers, the videos were rotated by [−30, −15, 0, 15, 30] degrees and inverted. Hence, for every video clip, we generated a further 8 video clips. By simulating different viewing angles and perspectives, the rotation and inversion of the videos aims to increase the classifier’s robustness and performance in classifying infant movements and postures. For NC, there were 5650 data samples; for FC1H, there were 3390 data samples; and for FC2H, there were 6630 data samples. For this work, and to maintain a class balance, FC2H and NC were randomly down-sampled to 3390 data samples.

### 2.1. Transformer Models and Diverse Video Features

To try and extract the most useful information contained within the video clips, this work utilises three different deep-neural-network-based models, feeding into a transformer network. For all these models, we removed the final layer and took the features derived from the models and use these as inputs to the transformers. The first model is vit-base-patch16-224-in21, a Vision Transformer (ViT) model pre-trained on ImageNet-21k (14 million images, 21,843 classes) at resolution 224 × 224 [34]. ViT is a technique that has begun to overtake the traditional CNN models in terms of performance, learning speed and efficiency [35].

The second model that is used is ConvNeXtBase [36], a pre-trained convolutional neural network (CNN) architecture designed to bring the advantages of recent innovations in ViTs back to convolutional networks. This architecture maintains the hierarchical feature extraction of traditional CNNs while enhancing performance and scalability, making it highly effective for image classification and other computer vision tasks.

**Figure 1:**
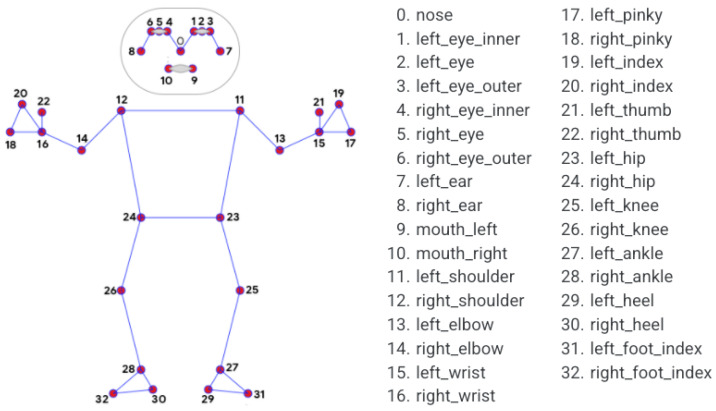
Each of the post estimation landmarks that Google’s MediaPipe produces. Each coordinate has an x, y, and z component (the z point is zeroed in 2D space), with a total 99 data points per image. The algorithm’s functionality is dependant on a clear, full view of the subject.

The third model that is used in Google’s Media pipe (Figure 1), a fast and accurate pose estimation algorithm. It provides a wide range of machine learning solutions for real-time media-processing tasks, such as hand and face tracking and object detection, and it has previously been shown to be effective in the pose estimation of children [33,37].

**Figure 2:**
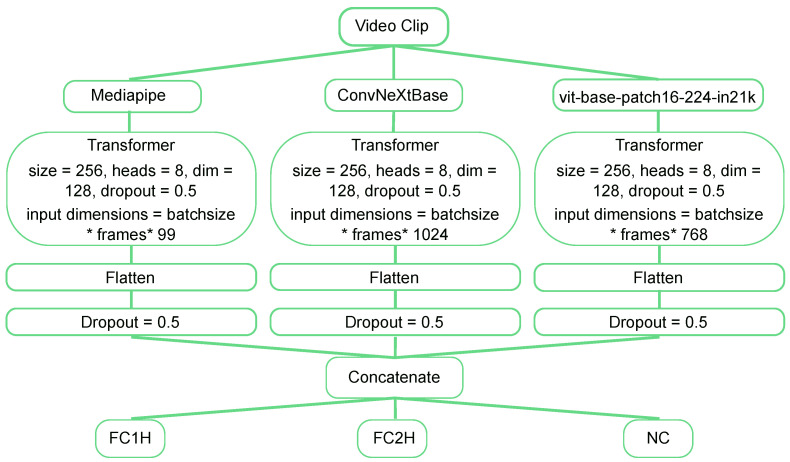
The network topology of the combined network is detailed, with results shown in Table 3 and the confusion matrix in Figure 3. Video clips were preprocessed by three networks (ViT, ConvNeXtBase, and MediaPipe) to improve training times by processing the data only once. The variation in input dimensions (ViT: 769, ConvNeXtBase: 1024, MediaPipe: 99) stems from the distinct architectures of each model. ViT processes image patches, yielding 769-dimensional embeddings, while ConvNeXtBase’s deeper layers result in 1024-dimensional features. MediaPipe, optimized for pose estimation, outputs a more compact 99-dimensional feature set. These differences reflect the specialized functions of each model. Additional dropout layers were used to prevent overfitting. The transformer encoder block is the same as that in the single-modality experiments and can be seen in Table 2; however, for the combined network, the dropout is 0.5 in comparison to 0.1 in the single networks.

In this work, the three algorithms were used separately to benchmark their performance in the network structure described in Table 1 and Table 2. The three networks were combined in the transformer-based fusion of diverse video feature model in Figure 2.

### 2.2. Deep Learning

For the first set of experiments, each of the pre-trained neural networks (ViT, ConvNeXtBase and MediaPipe) was used to create individual networks which fed directly into the transformer networks described in Table 1 and Table 2. Each of these networks was trained identically, where 70% of the data were used for training, and 15% each were used for validation and testing. There are 3390 samples per class, with 10,170 samples overall (class balanced, where the additional samples from FC2H and NC were randomly down-sampled), 7119 for training and 1525 each for validation and testing. The network optimiser used ADAM (adaptive moment estimation), and the network was trained for 1000 epochs, with two callback functions. The first is early stopping, which ceases training after the validation accuracy has not improved, with a patience of 20 epochs. In addition, there is a learning rate reduction, which, starting at 0.00001, was reduced by 10% over time with a patience of 20 epochs. There is an extensive use of dropout layers in the transformer networks to help prevent overfitting.

The second experiment combines the data from each of the three pre-trained networks into a single network as seen in Figure 2. The experimentation is identical to the above experiments where a single pre-trained network is used, except that it was found through exploratory expeditions that the combined model was more likely to overfit the data, so the dropout rate in the transformer block (Table 2) was increased from 0.1 to 0.5.

To improve the efficiency of training, the features derived from the pre-trained neural networks were computed separately prior from training, meaning that they only needed to be computed once, and not for each experiment. To total memory to store the preprocess features was 38.3 GB; hence, without preprocessing, the overheads would have been significant.

With the combined network being a complex architecture, sensitivity analysis was used to determine how the different features of data affect the performance and robustness of the network.This can help improve model simplification in later studies and identify where improvement could be made in terms of model performance [38,39].

## 3. Results

The primary aim of this work was to develop a robust and accurate transformer-based neural network comprising the fusion of diverse video features, which could ultimately perform well for the complex classification of motor tasks in infants. A further aim was to better understand how networks function, trying to improve their transparency and determine paths for future work.

The number of epochs for each of the training runs varies due to early stopping (Figure 4). In reference to this, all the training runs are close in terms of epochs, except for the MediaPipe only transformer network, which took around 60 epochs more to finish training, suggesting that the features take longer to learn. In all the experiments, the training data (solid line) were significantly more performant compared to the validation data (dashed line), which although expected to some extent, suggests that there may be some overfitting.

**Figure 3:**
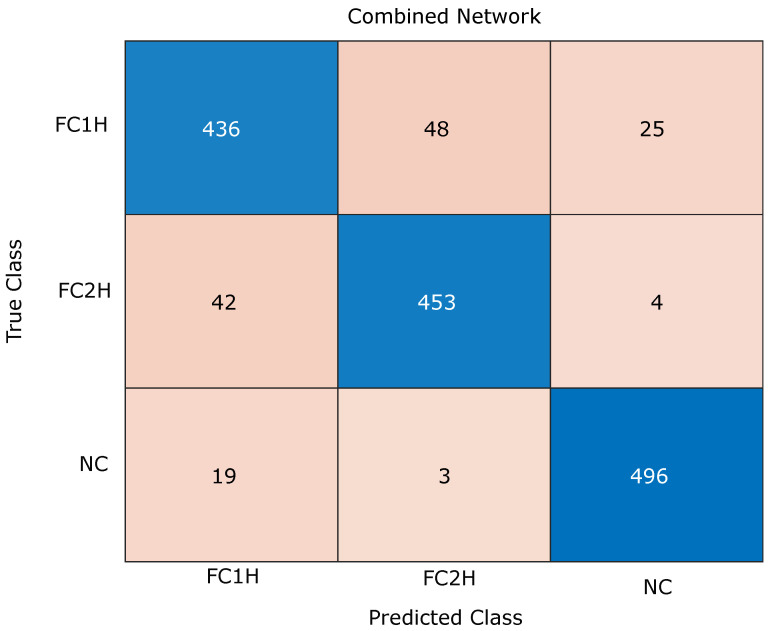
The confusion matrix for the combined experiment, with the results from the combined network in Table 3 with 90% accuracy. The results show that the NC class is the most accurately classified, and the FC1H and FC2H have more incorrect classifications, which was expected as they would appear to be more difficult to manually discriminate against when compared to NC.

**Figure 4:**
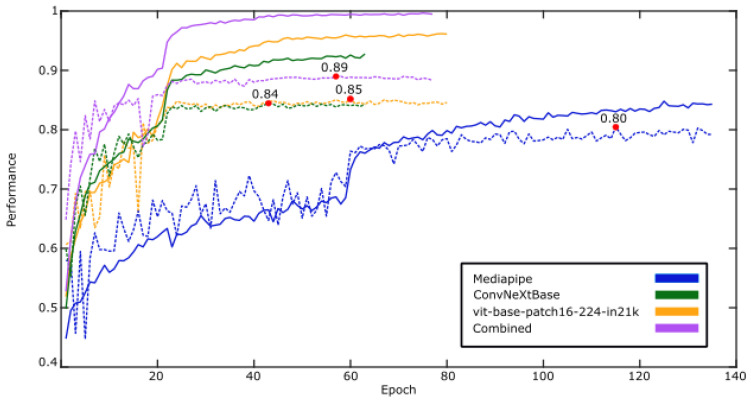
This figure shows the network’s overall training history for all modalities of data. The red dots mark the highest validation score, which triggered early stopping and the varying epochs. The MediaPipe-only transformer network required about 60 more epochs, indicating longer feature learning times. Training data (solid line) consistently outperformed validation data (dashed line), suggesting that there is some level of overfitting present.

The overall training of the network can be seen in Figure 4, and the overall results can be seen in Table 3. The three metrics in Table 3 are defined as follows: precision measures the proportion of correct positive predictions out of all positive predictions made by the model. It is calculated as $\frac{\mathrm{TP}}{\mathrm{TP}+\mathrm{FP}}$, where TP represents true positives and FP represents false positives. Recall, also known as sensitivity, measures the proportion of actual positive cases correctly identified by the model, calculated as $\frac{\mathrm{TP}}{\mathrm{TP}+\mathrm{FN}}$, where FN represents false negatives. Accuracy represents the overall correctness of the model, calculated as $\frac{\mathrm{TP}+\mathrm{TN}}{\mathrm{Total}\;\mathrm{Predictions}}$, where TN represents true negatives. What can be seen notably here is that there is a clear separation in the networks, with precision, recall, and accuracy not overlapping between the different networks. It can be seen that MediaPipe had the lowest performance with 78% accuracy. This might be because MediaPipe’s data contain less information than the other modalities, and it is prone to the loss of data from certain positions due to limb occlusions. Improving on this performance was vit-base-patch16-224-in21k, with 82% accuracy, followed by ConvNeXtBase with 84% accuracy. The features derived from these networks contain similar amounts of data with 769 and 1024 real values generated per frame, over 7 times that of MediaPipe. These results are summarised in the confusion matrix in Figure 3. The confusion matrix compares actual and predicted outcomes, with the diagonal (true positives and true negatives) showing correct predictions. Higher diagonal values indicate better performance.

The ConvNeXtBase network alone achieves an accuracy of 84%. However, when combining all three modalities in the integrated network (Figure 2), the accuracy exceeds 90%, marking a significant improvement of 6%. This suggests that the combined network enhances classification accuracy by effectively integrating and leveraging relevant features from the pre-trained networks.

### 3.1. Sensitivity Analysis

To generate an understanding of why the combined models are more performant over the single models, sensitivity analysis was performed. Sensitivity analysis is a technique used to determine how different variables in a model influence the output of that model. It involves systematically perturbing input variables to assess their effect on the outcome. To achieve this on the combined network, we perturbed each of the different modalities of data separately to gain an understanding of how each individual had an effect on the network.

**Figure 5:**
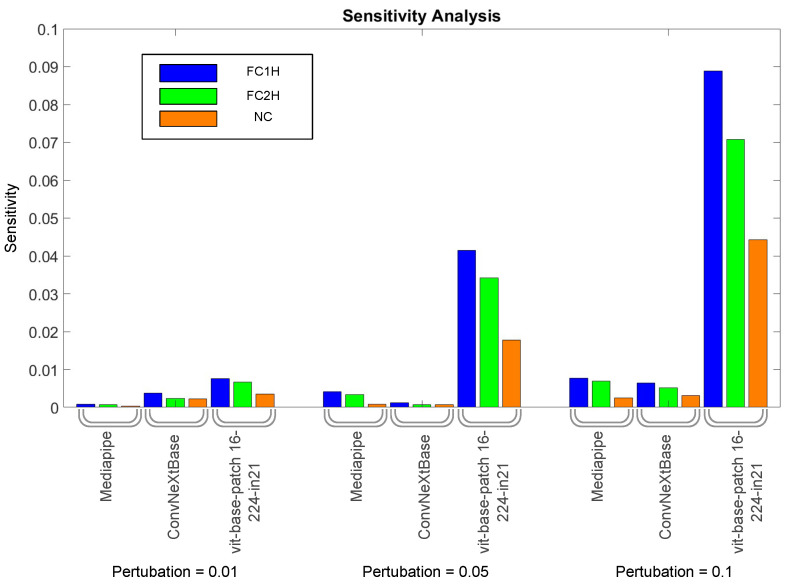
The sensitivity analysis broadly shows that larger perturbations cause larger effects on the outputs of the network, except for the case of ConvNeXtBase in-between the perturbations of size 0.01 and 0.05. The most striking trend in the data is that the vit-base-patch16-224-in21k is much more sensitive than the other modalities, signifying that the combined model is very dependant on vit-base-patch16-224-in21k. The change in sensitivity approximately trends linearly with the perturbation size.

The overall results of the sensitivity analysis can be seen in Figure 5. As the size of the perturbations increases, it can be seen that the sensitivity increases, except for the ConvNeXtBase between perturbation sizes of 0.01 and 0.05, which is an unexpected finding. More significantly, it can be seen that the vit-base-patch16-224-in21k network is the most sensitive to these perturbations; due to this, it is likely responsible for a larger proportion of the decision-making process, especially when the perturbation size increases.

The fact that the vit-base-patch16-224-in21k network is the most sensitive to perturbations in the combined network is somewhat surprising, given that it was not the top performer among the single-modality networks. However, it has the greatest influence on the combined network, indicating that the combined network relies heavily on vit-base-patch16-224-in21k for its decision-making process.

### 3.2. Interpretation of the Data

**Figure 6:**
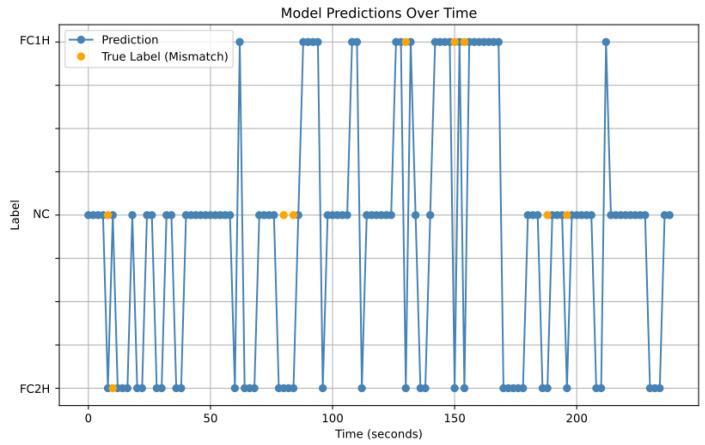
An example of how the data from a long video can be using in combination with the trained combined network to show behaviour over time. This is a 260-second video clip with an infant displaying FC1H (high line) and FC2H (low line), while also having rests from playing (middle line). The ground truth was highlighted where it differed from the predicted values. Such graphs can more accurately show behaviour over time, and they might be a useful tool for healthcare practitioners.

One of the difficulties of data-intensive work in a healthcare setting is to provide the results to healthcare professionals in a condensed but understandable manner. Due to the heterogeneity of infants, movement disorders and healthcare settings, it is important to view these data over longer time periods. To best show the results of a recorded session(s) overtime, it is best to combine the results into a frequency chart so that the aggregate behaviour can be seen over time. This can help remove variances due to the heterogeneity of the participants’ behaviour on a given day. An example of this for a single session form a single participant can be seen in Figure 6, which illustrates that the infant was consistently engaging with toys, in both FC1H and FC2H classifications, with periods of rest. Ideally, such results would be collated after multiple sessions to best show the data over wider time frames.

## 4. Discussion

It is important to acknowledge the limitations of this study. Firstly, the population size was limited to twelve parent–infant pairs, which may not represent broader populations in terms of socioeconomic, ethnic, and developmental diversity. This limited sample size could introduce bias and affect the generalizability of the results. While this study serves as an important proof of concept, future research with larger and more diverse populations is necessary to validate these findings and ensure their applicability across a wider range of infants.

Secondly, the data were collected at a single time point, which may not capture the full spectrum of individual variability. Factors such as an infant’s mood, health, or temporary behaviours during the recording session could influence the outcomes. Ideally, repeated assessments with the same individuals over time would provide a more comprehensive understanding of neurodevelopmental progress and allow the model to track individual developmental trajectories.

Future studies should focus on longitudinal data collection to account for these in-person variations and better identify developmental trends. Moreover, while this study focused on using machine vision to capture infant movements, we recognize the importance of parent–infant interactions in shaping motor behaviours. The current unstructured protocol allowed for natural exploration, but future research will incorporate more standardized parent–infant interaction protocols. This will help us better understand these influences and refine the accuracy of our machine vision models in neurodevelopmental assessment.

## 5. Conclusions

This study presents a transformer-based neural network model that successfully fuses multiple transformations of video data to classify infant interactions with toys, achieving an accuracy of 90%. The fusion model significantly outperformed individual component networks, illustrating the strength of combining features from various deep learning architectures. Notably, the sensitivity analysis revealed that the features extracted from the vit-base-patch16-224-in21k network were more crucial to the decision-making process than those from ConvNeXtBase and MediaPipe, despite not being the top-performing individual architectures in terms of accuracy. This finding highlights the critical role of diverse feature sets in enhancing model robustness and accuracy.

This work demonstrates the potential of using transformer-based models to accurately classify complex infant movements with just a single fixed-point camera, offering an improvement over traditional pose estimation methods. This approach reduces errors that often occur due to occluded limbs or obstructed poses. Additionally, the system’s low cost and scalability make it suitable for wider use, particularly in low-resource settings where early neurodevelopmental screening is crucial. Additionally, it could also enable parents to monitor their child’s development at home with minimal training, helping to identify potential delays or abnormal patterns in real time.

In conclusion, this work demonstrates the potential of transformer-based models in advancing neurodevelopmental diagnostics by leveraging feature fusion from multiple neural networks. Despite the limitations of sample size and single-session data, the model’s accuracy and cost-effectiveness suggest it could play a crucial role in developing scalable, global healthcare networks for early-stage neurodevelopmental screening and intervention. Future studies focusing on longitudinal data, larger populations, and multimodal data integration will be essential for realizing the full potential of this technology in improving neurodevelopmental outcomes worldwide.

## Figures and Tables

**Table 1:** The topology of the neural network structure when using a single modality of data. The network utilises the transformer model from Table 2.

Layer	Type	Parameters
Input	-	Input size (one of): ViT (49 × 769), ConvNeXtBase (49 × 1024), MediaPipe (49 × 99)
Transformer-Encoder	Transformer	head_size: 256, num_heads: 8, ff_dim: 128, dropout: 0.1
Flatten Layer 2	Flatten	-
Dropout	-	Rate: 0.1
Output	Dense	Units: 3, Activation: Softmax

**Table 2:** Transformer encoder structure which is part of the neural network structure from Table 1. This structure includes three dropout layers to best prevent overfitting.

Step	Layer	Parameters
1	Multi-Head Attention	key_dim: head_size, num_heads: num_heads, dropout: dropout
2	Dropout	rate: 0.5
3	Add	Adds input tensor and the output from Dropout
4	Dense	units: ff_dim, activation: ’relu’
5	Dropout	rate: 0.5
6	Dense	units: inputs.shape[−1]
7	Dropout	rate: 0.5
8	Add	Adds the final output and the tensor from step 3

**Table 3:** The results of the experiments conducted in this work are summarized as follows. The highest performing network using a single modality of data was ConvNeXtBase, achieving an accuracy of 84%. When combining multiple modalities, the overall network accuracy increased to 90.1%. The pose estimation algorithm, MediaPipe, produced the data with the lowest accuracy. This lower performance can be attributed in part to MediaPipe having approximately seven times less data compared to the other two modalities. Additionally, MediaPipe suffered from data loss when limbs were hidden in certain positions, which can be partially attributed to the fixed-point camera setup; this also highlights the general issue of limb occlusion in pose estimation. The confusion matrix showing the results from the combined experiment is shown in Figure 3.

	Precision	Recall	Accuracy
MediaPipe	0.7837	0.7788	0.7814
ConvNeXtBase	0.8388	0.8390	0.8404
vit-base-patch16-224-in21k	0.8237	0.8232	0.8228
Combined	0.9076	0.9069	0.9073

## Data Availability

Data are contained within the article.
